# Accuracy and Acceptability of the VISITECT CD4 Advanced Disease Test Compared With the PIMA CD4 Test at the Point of Care as Part of the Advanced HIV Disease Care Package: A Mixed-Methods Study

**DOI:** 10.1093/ofid/ofag043

**Published:** 2026-01-29

**Authors:** T Gils, S Misra, T Madonsela, T P Pita, S Bosman, I Ayakaka, E Vlieghe, A van Heerden, L Lynen, T Decroo, K Reither

**Affiliations:** Department of Clinical Sciences, Institute of Tropical Medicine, Antwerp, Belgium; University of Antwerp, Global Health Institute, Wilrijk, Belgium; Centre for Community Based Research, Human Science Research Council, Pietermaritzburg, South Africa; Wits Health Consortium, University of the Witwatersrand, Johannesburg, South Africa; Centre for Community Based Research, Human Science Research Council, Pietermaritzburg, South Africa; Centre for Community Based Research, Human Science Research Council, Pietermaritzburg, South Africa; Centre for Community Based Research, Human Science Research Council, Pietermaritzburg, South Africa; SolidarMed, Partnerships for Health, Maseru, Lesotho; University of Antwerp, Global Health Institute, Wilrijk, Belgium; Department of General Internal Medicine, Infectious Diseases and Tropical Medicine, University Hospital Antwerp, Edegem, Belgium; Centre for Community Based Research, Human Science Research Council, Pietermaritzburg, South Africa; Wits Health Consortium, University of the Witwatersrand, Johannesburg, South Africa; Department of Clinical Sciences, Institute of Tropical Medicine, Antwerp, Belgium; Department of Clinical Sciences, Institute of Tropical Medicine, Antwerp, Belgium; Department of Medicine, Swiss Tropical and Public Health Institute, Allschwil, Switzerland; University of Basel, Basel, Switzerland

**Keywords:** enhanced package of care, qualitative research, sensitivity, specificity

## Abstract

**Background:**

CD4 testing is the first step of the advanced human immunodeficiency virus (HIV) disease (AHD) care package. The semiquantitative VISITECT CD4 Advanced Disease test (VISITECT; AccuBio) is increasingly used but has variable reported performance. We compared its diagnostic accuracy and acceptability with that of the PIMA CD4 test (PIMA; Abbott) at the point of care within the AHD care package.

**Methods:**

A mixed-methods study was embedded in a community-based tuberculosis case-finding trial in South Africa. Following a VISITECT batch recall (April 2023), a sample of trial participants with HIV underwent VISITECT and PIMA testing in parallel on a single venous blood sample. The sensitivity, specificity, positive predictive value (PPV), and negative predictive value (NPV) were calculated. We conducted in-depth-interviews with people with HIV (PWH), focus group discussions with nurses, and participant observations with nurse-PWH pairs.

**Results:**

Among 609 included PWH, 76 (12.5%) were found to have CD4 cell counts ≤200/µL with VISITECT versus 28 (4.8%) with PIMA. The sensitivity, specificity, PPV, and NPV for VISITECT, compared with PIMA, were 89.3% (95% confidence interval, 71.8%–97.7%), 91.2% (95% CI, 88.6%–93.4%), 32.9% (95% CI, 22.5%–44.6%) and 99.4% (95% CI, 98.4%–99.9%), respectively. Three VISITECT results (0.05%) were false-negative (CD4 cell counts, 16/µL, 82/µL, 175/µL) and 51 (8.4%) were false-positive (median CD4 cell count, 563/µL [interquartile range, 382–739/µL]). PWH and public sector nurses believed that point-of-care CD4 tests improve access to CD4 cell counts. Study nurses preferred PIMA to VISITECT in terms of accuracy, duration, user-friendliness, and yielding of a numeric CD4 result.

**Conclusions:**

Compared with PIMA, VISITECT had good diagnostic accuracy but low PPV and was less acceptable to study nurses. The role of VISITECT should be further evaluated.

## Teaser

The VISITECT CD4 Advanced Disease test had good sensitivity and specificity but low positive predictive value compared with the PIMA CD4 test, when performed at the point of care in the community, within an advanced human immunodeficiency virus care package. Study nurses preferred PIMA to VISITECT.

An estimated 630 000 individuals died of AIDS-related illness in 2024 [1]. Globally, about 30% of people with human immunodeficiency virus (HIV; PWH) enter or reenter care with advanced HIV disease (AHD) [2]. AHD is associated with elevated mortality rates, mainly from tuberculosis, other invasive bacterial infections, and cryptococcal meningitis, conditions requiring urgent medical intervention [3–5]. AHD is defined in those >5 years old as a World Health Organization (WHO) clinical stage 3 or 4 condition or a CD4 cell count <200/µL [6].

CD4 determination is critical to identify AHD, as clinical staging has a low sensitivity at CD4 cell counts <200/µL [4]. After implementation of a universal test and treat approach and because viral load is used to monitor antiretroviral treatment (ART) progress, measuring CD4 cell counts has become less common at public health facilities [6, 7]. In many countries depending on external HIV funding, limited funding was shifted away from CD4 testing [8]. The consequent decline in global demand for CD4 testing led 2 principal CD4 device manufacturers to halt production of CD4 testing devices for commercial reasons, Becton Dickinson Biosciences (BD) withdrew BD FACScount and FACSpresto and Abbott its point-of-care (POC) PIMA CD4 analyzer (PIMA; Abbott). PIMA cartridges remain available for existing devices [6].

A package of care including tests and (prophylactic) treatment can reduce the mortality rate for AHD [9]. The lack of CD4 testing options hampers implementation of the AHD care package in many settings with a high HIV burden [10, 11]. The instrument-free semiquantitative POC VISITECT CD4 Advanced Disease test (VISITECT; AccuBio), differentiating between CD4 cell counts >200/µL or ≤200/µL, is increasingly used [12]. While VISITECT implementation was considered feasible, its usability and diagnostic performance vary in published studies (Supplementary Table 1) [12–18].

The diagnostic performance of VISITECT, as part of a community-delivered AHD care package, has not yet been evaluated, and healthcare worker preferences between VISITECT and the PIMA have not yet been qualitatively assessed. We compared the diagnostic accuracy of VISITECT with that of PIMA when performed at POC within the AHD care package, during community-based tuberculosis screening, and we evaluated the feasibility and acceptability of VISITECT and PIMA use among PWH and nurses.

## METHOD

### Design and Period

This mixed-methods study was embedded in a community-based prospective active tuberculosis triage study conducted between September 2022 and August 2024 in Lesotho and South Africa (TB TRIAGE+ TRIAL; Clinicaltrials.gov NCT05526885). Data for the current study were collected between April 2023 and June 2024.

### Setting

The study setting was Greater Edendale Area, Msunduzi Municipality, KwaZulu Natal Province, South Africa. The estimated HIV prevalence in that area was 36% in 2014–2015 [19]. Between 2020 and 2022, a feasibility study for implementing the AHD care package was embedded in the diagnostic accuracy study (TB TRIAGE+ ACCURACY) preceding the TB TRIAGE+ TRIAL in this setting [20, 21]. Then, we identified 29.0% (99 of 341) of PWH with AHD (CD4 cell count ≤200/µL with VISITECT or a tuberculosis diagnosis) among study participants with tuberculosis symptoms recruited near a health facility [22]. Currently, all public sector CD4 testing in South Africa is performed in centralized laboratories.

### Study Population

For the TB TRIAGE+ TRIAL, consenting adults were eligible. Exclusion criteria included requiring immediate medical care or being on antituberculosis treatment [23]. VISITECT was originally used as the CD4 test for all PWH. In April 2023, the VISITECT batch used since September 2022 was withdrawn by AccuBio following a specificity drop below 70%. Following the recall, and as preliminary data from another study suggested suboptimal specificity with VISITECT [24], we replaced VISITECT with PIMA as the reference CD4 test. In consecutively recruited PWH (from September 2023 to May 2024) VISITECT was used in parallel to PIMA. PWH who received both tests were included for quantitative data collection, until the sample size for this study was reached.

In-depth-interviews (IDIs) were performed with PWH who had experienced VISITECT and/or PIMA testing, aiming for a majority with AHD. All study nurses who implemented AHD procedures, and invited public sector nurses from surrounding clinics were included in focus group discussions (FGDs). Study nurse–PWH pairs performing/receiving CD4 testing were eligible for participant observations (POs).

### Study Procedures

#### Trial Procedures

Detailed trial procedures were described elsewhere [23]. Consenting individuals identified with tuberculosis screening tests (ie, Computer-Aided Detection for Tuberculosis [CAD4TBv7] or CAD4TBv7 with C-reactive protein) were tested with the Xpert MTB/RIF Ultra (Cepheid) [23]. PWH underwent a CD4 test and, if the CD4 cell count was ≤200/µL, a Determine tuberculosis lipoarabinomannan (LAM) urine test (TB LAM; Abbott) and a plasma cryptococcal antigen (CrAg) test (Immy). Same-day initiation of ART, tuberculosis-preventive therapy and cotrimoxazole were provided per guidelines. Quantitative data were immediately entered with Research Electronic Data Capture (REDCap) tools on tablets.

#### Index and Reference Testing

VISITECT was performed by nurses per the manufacturer's instructions. A venous blood sample was collected via phlebotomy in an ethylenediaminetetraacetic acid tube. After the tube was gently inverted at least 8 times, 30 µL was pipetted and used for the VISITECT test. The sample was added to well A. After 3 minutes, 1 drop of buffer was added to well A. After 17 minutes, 3 drops of buffer were added to well B. After an additional 20 minutes, results were interpreted by the nurse by comparing the color intensity of the test line to a 200-reference line on the test device. In the absence of a control line or a 200-reference line, the test was declared invalid and repeated. Pictures were collected of all VISITECT tests at the time results were read. Results were entered into REDCap.

PIMA was performed per the manufacturer's instructions by another nurse in another room, on the same venous blood sample used for VISITECT [24]. Daily PIMA calibrations were performed. Nurses were blinded to the results of the other test while performing and reading the tests. The PIMA result was communicated to and captured in REDCap by the nurse performing VISITECT. In case of a PIMA CD4 cell count ≤200/µL, the TB LAM and CrAg test were performed.

For a subsample of consecutively recruited participants, a trained data collector conducted a second VISITECT reading, blinded to the PIMA result and the first VISITECT reading. The reading was captured separately by the second reader on a paper-based log and later entered in Microsoft Excel.

#### Qualitative Methods

A qualitative study embedded in the TB TRIAGE+ TRIAL assessed the feasibility of implementing the AHD care package. During 2 FGDs with study nurses and 2 with public sector nurses, participants were asked about their experiences with VISITECT and/or PIMA, their preferences, and the acceptability of both tests. Public sector nurses received a presentation about the AHD care package before the FGD. IDIs were conducted with PWH to assess the acceptability of AHD care package procedures for them, which included VISITECT and/or PIMA. POs were conducted during implementation of the AHD care package to further observe its acceptability for nurses and PWH and challenges with implementation. All qualitative procedures were conducted by trained qualitative researchers, aided by a pretested topic guide. IDI and FGD data were first recorded in audio form, after which they were transcribed and translated on Microsoft Word documents. A demographic form and a memo for IDIs and FGDs were captured in Microsoft Word.

### Outcomes

For VISITECT, we estimated the sensitivity, specificity, positive predictive value (PPV), and negative predictive value (NPV) (with 95% confidence intervals [CIs]) for identifying a CD4 cell count ≤200/µL, compared with PIMA. We calculated the proportion of PWH in whom VISITECT misclassified the CD4 cell count, overall and by different CD4 cell count strata (0–100/µL, 101–200/µL, 201–350/µL, 351–500/µL, and >500/µL). In a subgroup, we calculated the proportion of result agreement between 2 VISITECT readers. Post hoc, we assessed the adjusted odds ratios (aORs) for predictors of VISITECT positivity. Qualitative outcomes included perceptions of the acceptability and feasibility of implementing VISITECT and PIMA.

### Sample Size

To establish sensitivity and specificity (95% CI; maximum width, 0.1; α = 0.05; power, 80%), assuming a maximum of 7.8% of PWH with a CD4 cell count ≤200/µL (identified by VISITECT in early trial data) and a minimum of 3.9% (expected with PIMA) and anticipating a minimum specificity of 60% and a minimum sensitivity of 94% [13, 14, 24], the minimum inclusion was 556 PWH. Adding 10% missing data, we aimed to recruit 611 PWH. To estimate the interrater agreement, assuming an expected 90% results concordance (95% CI; maximum width, 0.1; α = .05; *z* = 1.96; δ = 0.05), we needed 139 second readings. For qualitative data, we aimed to recruit until saturation, to a maximum of 15 PWH for IDIs, 10 study nurses and 10 public sector healthcare workers for FGDs. and 6 study nurse–PWH pairs for POs.

### Analysis

Categorical variables were presented with frequencies, proportions, and 95% Clopper-Pearson CIs for the main outcomes, and continuous variables with medians and interquartile ranges. We calculated the Cohen κ coefficient for interrater agreement between 2 VISITECT readers. Predictors for VISITECT positivity were explored by including all variables of interest in a multivariable logistic regression model, including the CD4 measured on PIMA. We considered differences to be statistically significant at *P* < .05. Analyses were done using Stata software, version 16.1 (StataCorp).

Findings relevant to CD4 testing in IDIs, FGDs, and POs were coded by 2 researchers in parallel using NVivo software, version 1.7 (QSR International), and final codes were agreed upon by a third researcher. Thematic analysis was used to categorize identified codes into broader themes. We triangulated quantitative and qualitative data.

### Ethical Approval

This study and the TB TRIAGE+ TRIAL received ethics approval by the Human Sciences Research Council Research Ethics Committee (3/18/01/23 and 2/23/09/20) and the Ethikkomission Nordwest-und Zentralschweiz, Switzerland (AO_2023-00014) and support from the Provincial Department of Health, KwaZulu Natal, South Africa [23]. Study participants provided written informed consent for quantitative and qualitative data collection separately.

## RESULTS

### Inclusion and Characteristics of People With HIV

The TB TRIAGE+ TRIAL included 6987 persons in South Africa, of whom 1968 had HIV (28.2%). Among them, 940 (47.8%) had VISITECT results, 941 (47.8%) had PIMA results, and 609 (31.0%) had both CD4 test results (Table 1).

**Table 1.** Characteristics and Test Results of People With Human Immunodeficiency Virus and With VISITECT and PIMA CD4 Results (N = 609)

Characteristics and Results	PWH, No. (%)^a^
Age, median (IQR), y	42 (34–51)
Sex	
Female	423 (69.5)
Male	185 (30.4)
Ambiguous/intersex	1 (0.2)
HIV/ART status	
Known HIV positive on ART	592 (97.2)
Known HIV positive not on ART	6 (1.0)
HIV newly diagnosed	11 (1.8)
VISITECT result, CD4 cell count	
0–200/µL	76 (12.5)
>200/µL	533 (87.5)
PIMA result, CD4 cell count, median (IQR)	637/µL (443–834/µL)
PIMA CD4 cell count category	
0–200/µL	28 (4.6)
>200/µL	581 (95.4)
TB LAM result	
Positive	0 (0.0)
Negative	28 (4.6)
Not eligible^b^	581 (95.4)
CrAg result	
Positive	0 (0.0)
Negative	28 (4.6)
Not eligible^b^	581 (95.4)

Abbreviations: ART, antiretroviral treatment; CrAg, cryptococcal antigen test (Immy); HIV, human immunodeficiency virus; IQR, interquartile range; PIMA, PIMA CD4 test (Abbott); PWH, people with HIV; TB LAM, Determine tuberculosis lipoarabinomannan antigen test (Abbott); VISITECT, VISITECT CD4 Advanced Disease test (AccuBio).

^a^Data represent no. (%) of PWH unless otherwise specified.

^b^CD4 cell count >200/µL by PIMA test.

### Diagnostic Accuracy

The CD4 cell counts measured by VISITECT compared with PIMA are shown in Table 2. The sensitivity for VISITECT was 89.5% (95% CI, 71.8%–97.7%) and the specificity was 91.2% (95% CI, 88.6%–93.4%). The PPV and NPV were 32.9% (95% CI, 22.5%–44.6%) and 99.4% (95% CI, 98.4%–99.9%), respectively. Fifty-four persons (8.9%) were classified differently by VISITECT than by PIMA; 3 (0.05%) false-negatives (CD4 cell counts, 16/µL, 82/µL, and 175/µL) and 51 (8.4%) false-positives (median CD4 cell count, 563/µL [interquartile range, 382–739/µL) for detection of a CD4 cell count ≤200/µL. Among persons with PIMA CD4 cell counts 201–300/µL, 301–500/µL, or >500/µL, respectively, 4 of 27 (14.8%), 18 of 143 (12.6%), and 29 of 411 (7.1%) were classified as positive (≤200/µL) with VISITECT.

**Table 2.** Comparison of VISITECT and PIMA CD4 cell counts

VISITECT Result, CD4 Cell Count	PIMA Result, CD4 Cell Count		
	≤200/µL	>200/µL	Total
≤200/µL	25	51	76
>200/µL	3	530	533
Total	28	581	609

Abbreviations: PIMA, Abbott PIMA CD4 test (Abbott); VISITECT, VISITECT CD4 Advanced Disease test (AccuBio).

### Interrater Agreement

In 12 of 183 VISITECT tests (6.6%) read by a second reader, there was reader disagreement, equivalent to 93.4% agreement (κ = 0.6776; *P* < .00). Examples of VISITECT readings with an interrater disagreement are shown in Supplementary Figure 1.

### Comparison With Existing Evaluations of VISITECT Accuracy

**Figure 1.**
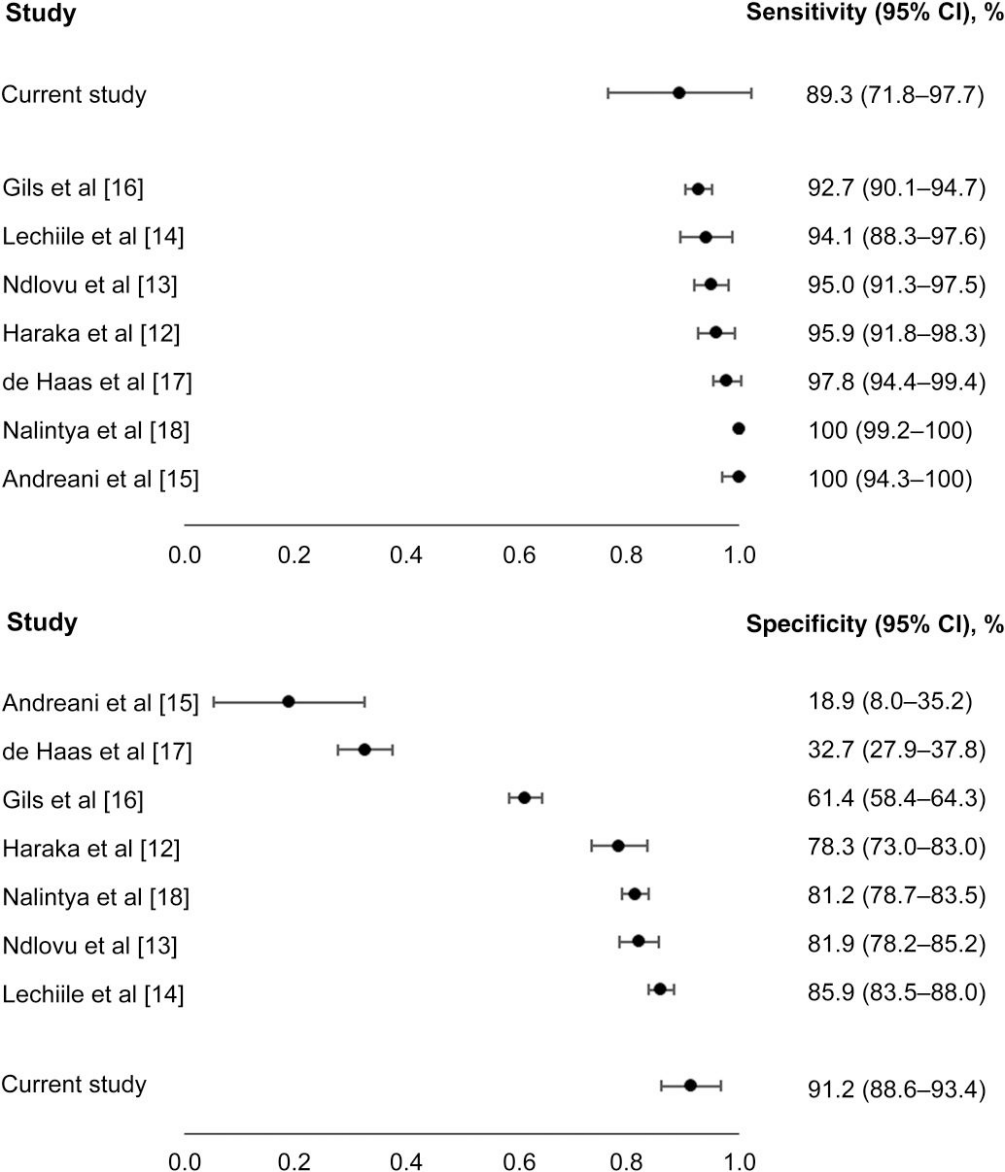
Forest plot of the sensitivity and specificity of the VISITECT CD4 Advanced Disease test (VISITECT; AccuBio) in the current study, compared with findings in published articles [12–18]. Abbreviation: CI, confidence interval.

Figure 1 graphically presents VISITECT sensitivity and specificity estimated in this study, compared with previously publications [12–18].

### Predictors of VISITECT Positivity

In a multivariable logistic regression model, including CD4 measured by PIMA, the time since study start (aOR, 0.99 [95% CI, .99–1.00]) was associated with lower odds of a positive VISITECT result, while being male or ambiguous/intersex (vs female; aOR, 1.87 [95% CI, 1.04–3.35]) and using VISITECT batch Y (vs X; aOR, 6.38 [95% CI, 1.88–21.6]) were associated with higher odds (Supplementary Table 2).

### Characteristics of Qualitative Study Population

Saturation was reached after 13 IDIs with PWH. We conducted 2 FGDs with 5 study nurses in April 2023 (just after the VISITECT batch recall, FGD1), and 6 in March 2024 (during PIMA and VISITECT comparison, FGD2) (3 nurses participated twice). Two FGDs (FGD3 and FGD4) with public sector nurses included 4 and 3 participants from 2 clinics (Table 3).

**Table 3.** Characteristics of Participants in In-Depth Interviews and Focus Group Discussions

Characteristics^a^	Participants, No. (%)^b^
PWH (n = 13)	
Female sex	6 (46)
Age median (IQR), y	45 (41–49)
Highest educational level	
Primary school	3 (23)
Secondary school	1 (8)
High school	8 (62)
Tertiary or above	1 (8)
Employment status	
Unemployed	12 (92)
Employed in trade/sales	1 (8)
HIV status	
HIV newly diagnosed	2 (15)
Known HIV positive on ART	10 (77)
Known HIV positive not on ART	1 (8)
PIMA CD4 cell count, median (IQR)	157/µL (144–350/µL)
PIMA CD4 cell count ≤200/µL (n = 12)	8 (67)
VISITECT CD4 cell count ≤200/µL	13 (100)
AlereLAM positive (n = 9)	0 (0)
Xpert positive (n = 6)	0 (0)
CrAg positive (n = 9)	1 (11)
Nurses (n = 14)	
Study nurses	7 (50)
Public sector nurses	7 (50)
Female sex	12 (86)
Age, median (IQR), y	44 (37–51)
Tertiary education or above	14 (100)
Experience as a nurse, median (IQR), y	16 (10–20)
Experience with TB LAM^c^	9 (64)

Abbreviations: TB LAM, Determine tuberculosis lipoarabinomannan (LAM) antigen test (Abbott); ART, antiretroviral therapy; CrAg, cryptococcal antigen test (Immy); HIV, human immunodeficiency virus; IQR, interquartile range; PIMA, PIMA CD4 test (Abbott); PWH, people with HIV; VISITECT, VISITECT CD4 Advanced Disease test (AccuBio); Xpert, Xpert MTB RIF Ultra (Cepheid).

^a^All participants were South African, of African ethnicity and with IsiZulu as their first language.

^b^Data represent no. (%) of participants (PWH or nurses) unless otherwise specified.

^c^Previous to the TB TRIAGE+ ACCURACY study. None had experience with VISITECT or CrAg.

### Qualitative Analysis results

An selection of emerging themes and quotes related to CD4 testing is shown in Table 4.

**Table 4.** Selection of Themes Related to CD4 Testing Emerging From Qualitative Analysis

Themes	Subthemes	Representative Quotes	Person and Source
Accuracy and trust in test results	Accuracy	“VISITECT is a problem my God! You find that its results are not the same as the PIMA ones. It will give results that are below 200 whereas PIMA will produce 1000 and more! You don’t know how to explain to [the PWH], it doesn’t seem trustworthy that's its problem.”	Female study nurse (aged 36 y, 11 y experience) (FGD2)
		“The whole package is okay but the tests, if they were to change, take out VISITECT and maybe put another test, maybe PIMA CD4. Then we will know that we are working with something accurate that will help the participants in the right way.”	Male study nurse (aged 38 y, 16 y experience) (FGD1)
	Loss of trust	“Ey, we will do it [VISITECT testing] because the protocol forces us to. But if it were up to me, if I had a choice, no, I wouldn’t do it. I would not do it shame! I would rather choose … if we could do the normal CD4, I would go for that”	Female study nurse (aged 39 y, 10 y experience) (FGD1)
	Patient mismanagement	“A person who has been decanted by the clinic must fetch their medication somewhere else and not in the clinic … those people have stable blood because they get tested for bloods at the clinic. So, you can't tell me, that a person who fetches their pills at the pharmacy, and then when they come here, we say their CD4 is below 200.”	Female study nurse (aged 39 y, 10 y experience) (FGD1)
		“I would say there was a lot of mismanagement of the participant due to VISITECT not being able to give accurate results, so now we are being assisted by PIMA. We can say that PIMA is more accurate than VISITECT.”	Female study nurse (aged 58 y, 20 y experience) (FGD2)
Usability	Reading results	“When the results come out, they are not clear. You have to communicate with someone else for them to verify that are you really seeing two lines here or not. So, nothing is clear, maybe if the results are positive, maybe the colour must be a different colour or something.”	Female study nurse (aged 39 y, 10 y experience) (FGD1)
	Complexity of procedure	“It's not okay when you do it. The steps that are there … because if you make a small mistake then oh, those results are invalid. You will just interpret something that is not correct. Yeah, just the steps that are there … they are very complicated, and they take a long time!”	Female study nurse (aged 36 y, 11 y experience) (FGD1)
		“So, it would be better if you put the blood then wait and read the results after. But, no, you have to put, set a timer for 3 minutes, put another thing, set another timer for 17 minutes, put another thing and then time again for 20 minutes. You also time the 5 minutes at the end. So, you're timing every step of the way, you’re busy timing non-stop. I think that's where it gets problems a lot, a lot!”	Female study nurse (aged 39 y, 10 y experience) (FGD1)
	Not an exact CD4 cell count	“At the clinics when they do CD4 counts, they tell you the exact numbers … So, for us, the fact that we can’t even give people their CD4 count number, it's a problem because they end up not understanding well. When I say it's below, or when I say its above, how high is it?”	Female study nurse (aged 39 y, 10 y experience) (FGD1)
		“If I do it with PIMA it will tell me that your CD4 count is 190 then we will know how much it is rather than [with VISITECT]. So, you will also be able to take care of yourself even much better.”	Female study nurse (aged 39 y, 10 y experience) (FGD1)
	Test duration	“At the clinics they have a lot of headcount. Plenty of people are seen, can you imagine the 40 minutes, the hour that you will spend with a patient? If there are 50 people that you need to see, it's not possible for time (aged we should take out VISITECT. Nobody consults for 40 minutes!”	Male study nurse (aged 40 y, 17 y experience) (FGD2)
Integration into the healthcare system	Availability	“The Abbott company, do they still manufacture PIMA? If not, it would be a big problem because if PIMA gets left behind, VISITECT will also get left behind.”	Male study nurse (aged 40 y, 11 y experience) (FGD2)
	Training others	“The problem would be, will they [other healthcare workers] accept it? Problems will still be the same when you have taught them because the problem is not teaching them, the problem is with VISITECT.”	Female study nurse (aged 58 y, 20 y experience) (FGD1)
PWH perceptions	Current clinic practice	“What can they say when they are rushing to finish? If they take blood in December, they don’t come back with the right results that they will put in your file!”	Male PWH (aged 64 y, known HIV positive on ART, AHD) (IDI)
	Test duration	“VISITECT, if only they could make it fast so that you quickly know. Do you know how painful it is going to sleep panicking that ‘Eish, I wonder what the results will come back saying.’”	Female PWH (aged 26 y, HIV positive on ART, AHD) (IDI)

Abbreviations: AHD, advanced HIV disease; ART, antiretroviral treatment; FGD, focus group discussion; HIV, human immunodeficiency virus; IDI, in-depth-interview; PWH, people (or person) with HIV.

#### Accuracy and Trust in results

VISITECT was not acceptable to study nurses after the batch recall, as they had lost trust in its accuracy. They were adamant about removing VISITECT, and replacing it with, for example, PIMA. They worried about the impact of giving incorrect results to PHW and clinical management with negative consequences based on the results: “There are people who are on cotrimoxazole now who don’t need to take it. That is contributing to their liver toxicity” (female study nurse, aged 39 years with 10 years of experience; FGD1).

After PIMA introduction, differences in results with VISITECT were confirmed. Nurses felt uncomfortable showing VISITECT results to patients during the time both tests were used: “Yes, it is sometimes untrustworthy, you end up reading the [VISITECT] results with depression because you don’t know how to explain but then you do explain that ‘we will proceed with this one, yes, PIMA’” (female study nurse, aged 36 years with 11 years of experience; FGD2)

#### Usability

Study nurses highlighted multiple challenges when using VISITECT. They considered that the inaccuracy of VISITECT resulted in part from their own difficulties with reading results or procedural complexity: “The steps that are there … because if you make a small mistake then oh, those results are invalid. You will just interpret something that is not correct*”* (female study nurse, aged 36 years with 11 years of experience; FGD1) and “Our eyes will never be the same, if I take a quick look and see it [the test line] as light, then everyone will have AHD …” (female study nurse, aged 39 years with 10 years of experience; FGD3). The fact that VISITECT does not produce a numeric CD4 cell count was considered an inadequacy by nurses, and the ability of PIMA to do so was considered a superior quality.

#### Integration Into the Healthcare System

Public sector nurses were excited to use POC CD4 testing devices in the future to reduce the results turnaround time and clinic contact points for patients. They thought that this would improve patient visit compliance. A current lack of clinic information on CD4 results was confirmed by PWH. Public sector nurses thought that CD4 testing would be best delivered at primary clinics or outreach activities.

The long duration of the VISITECT procedure (>40 minutes) was seen as a problem by some patients and nurses, especially considering implementation in public clinics with high patient burdens. The duration of PIMA was considered better, making it more feasible to implement. However, study nurses were worried about PIMA availability, having heard about its market withdrawal.

#### Perceptions of CD4 Testing Among PWH

PWH were happy with the CD4 tests, and especially satisfied with community delivery of the AHD care package. Some mentioned that they would like to receive it at (mobile) clinics, community halls, local shops, or home. Some PWH were confused that they did not receive an exact CD4 cell count after VISITECT: “They told me that my CD4 cells are below 200. They have to be 200 but they were, what? 153, 137…? so it was dropping” (male person with HIV, aged 64 years; known to be HIV positive on ART with AHD; IDI).

## DISCUSSION

We compared VISITECT with PIMA, implemented at POC within the AHD care package, during community-based tuberculosis screening. VISITECT had an acceptable specificity of 91.2% (95% CI, 88.6%–93.4%) and sensitivity of 89.3% (95% CI, 71.8%–97.7%). While the NPV was optimal, the PPV was low. Both tests were acceptable to PWH and to public sector nurses without previous access to POC CD4. Study nurses strongly preferred PIMA in terms of accuracy, producing a numeric CD4 cell count, and test duration.

In peer-reviewed studies, the sensitivity of VISITECT varied between 93% and 100%, and the specificity between 19% and 86% [12–18]. In comparison, we found a lower sensitivity and a higher specificity. We used the same sample for both tests, eliminating intrasample variation. If different samples are used, it is possible to have a “true” positive and “true” negative sample from the same person, when values approach the threshold. PIMA is not the gold standard for CD4 testing, although it was used to measure VISITECT performance against in this study. With venous blood, PIMA sensitivity varied between 83% and 98% and specificity between 94% and 98%, compared with flow cytometry, at a CD4 cell count cutoff of 200/µL [26]. However, in a recent study the reported sensitivity and specificity of PIMA with finger-stick were 56% and 98%, respectively, compared with flow cytometry [25]. Our results could differ if flow cytometry were used as the reference. Given the consistent high sensitivity of VISITECT, and if indeed PIMA sensitivity was suboptimal, VISITECT may have correctly detected some “missed positives” by PIMA, in which case our observed VISITECT sensitivity is underestimated. If the specificity of PIMA is high, this is unlikely to introduce a strong bias in our estimation, while if it is suboptimal, we may have underestimated the specificity VISITECT in this study. Generally, an imperfect reference attenuates both accuracy measures. If the specificity of VISITECT was indeed truly better than in other studies, the nurses' experience in 2 large trials and repeated training may have contributed.

In previous studies, most misclassification by VISITECT happened with PIMA CD4 cell counts of 200–300/µL [13, 14, 16]. We also observed the highest proportion (14.8%) of false-positive VISITECT results in that range. However, the median CD4 cell count among the false-positives was 563/µL. In another study, VISITECT misclassified 48% of those with flow cytometry CD4 cell counts of 300–500/µL and 23% of those with counts >500/µL as having a CD4 cell count ≤200/µL [16].

The PPV of VISITECT was only 32.9% in our study; only 1 in 3 samples with positive VISITECT results was also positive on PIMA. As the PPV is dependent on AHD prevalence, the low observed prevalence (4.6%) contributed to this result. The PPVs would have been 52% and 72%, respectively, in scenarios with a 10% or 20% prevalence. Since PHW who were receiving tuberculosis treatment or were seriously ill were excluded from the study, the true population prevalence is likely higher [23].

We found substantial agreement (93.4%) between VISITECT readers, within the range of previously reported data (ie, 85.2%–97.5% agreement) [13, 14]. However, we confirmed with quantitative and qualitative data that VISITECT results are often hard to interpret, leading to interreader variation and deviations from the reference CD4 result [13, 22].

As in our study, variation in VISITECT positivity by VISITECT batch number and higher odds of VISITECT positivity in males were previously reported [16]. As VISITECT results are prone to interpretation, male PWH, who are more likely to have AHD, may be more likely than female PWH to be classified with a CD4 cell count ≤200/µL [26]. We also found variation in VISITECT positivity by the time between the start of the study and the performance of the test, which may reflect, for example, changes in operator performance (not evaluated), VISITECT batch (partly unaccounted for due to missingness), or a decrease in test stability. The subjectivity associated with VISITECT results interpretation likely also contributed to false-positives with high CD4 cell counts.

PWH and public sector nurses accepted both tests. Study nurses could no longer accept the long procedure, difficulties in reading results, and the lack of a numeric CD4 cell count after knowing about the low of accuracy VISITECT, exposed by the batch recall. The lack of a numeric CD4 cell count has clinical implications, as management may differ for thresholds other than 200/µL [22, 27].

Our study has limitations. We are uncertain to which extend the imperfect reference influenced our results. Study nurses were influenced by the batch recall, despite the good diagnostic accuracy observed in this study. Our multivariable analysis was limited by missing batch numbers, and there are factors unaccounted for since the study was not designed to evaluate predictors. Strengths of our study include the mixed-methods approach, allowing data triangulation and solid data collection procedures within a clinical trial but with pragmatic POC CD4 test implementation in the community, the test's intended use.

While WHO has reaffirmed the crucial role of CD4 testing to tackle AHD, the CD4 testing crisis persists due to product withdrawal and growing evidence of varying VISITECT performance [6]. Laboratory-based CD4 testing options are limited to Aquios (Beckman Coulter) and Sysmex Partic. While PIMA cartridges remain available for a device-based POC option, new devices are no longer manufactured, limiting this option in time [28]. One novel portable POC enumeration test, the VITA CD4 analyzer (VITA; Accesso Biotech), showed promising diagnostic performance but is not yet commercially available [29]. With no better options within reach, the use of VISITECT should be optimized.

An ongoing systematic review and meta-analysis will further evaluate factors contributing to VISITECT performance [30]. Artificial intelligence tools for the automated reading of visual tests may improve reading quality for VISITECT [31]. The WHO targets for a POC CD4 test to identify PWH with AHD are a minimum sensitivity and specificity of 80% [32]. Given its high sensitivity but varying specificity, VISITECT may be better placed as a CD4 triage test, but only if a more specific CD4 test is available. One study estimated the cost of using VISITECT before CrAg and TB LAM screening in a high-prevalence setting (>30% with CD4 cell counts ≤200/µL), equal to omitting VISITECT and testing everyone with a CrAg test and TB LAM [18]. If venous blood sampling was used for VISITECT, this would not change the number of blood samples per person with HIV, but it would increase the number of urine samples and the workflow. A head-to-head evaluation of VISITECT, PIMA, and VITA versus flow cytometry under alternative testing algorithms (including CD4 omission and CD4-based triage) would offer actionable evidence to guide implementation and policy decisions.

Compared with those of PIMA, the sensitivity and specificity of VISITECT were good during pragmatic community implementation within an AHD care package. However, its PPV was low in this setting with low AHD prevalence, and PIMA was considered more acceptable to users. The role of VISITECT as part of different algorithms should be evaluated. Novel, accurate, and user-friendly POC CD4 tests are needed.

## Supplementary Material

ofag043_Supplementary_Data

## Supplementary Data

Supplementary materials are available at *Open Forum Infectious Diseases* online. Consisting of data provided by the authors to benefit the reader, the posted materials are not copyedited and are the sole responsibility of the authors, so questions or comments should be addressed to the corresponding author.
