# Supplementary material for: Accuracy and Acceptability of the VISITECT CD4 Advanced Disease Test Compared With the PIMA CD4 Test at the Point of Care as Part of the Advanced HIV Disease Care Package: A Mixed-Methods Study
Source: Open Forum Infect Dis. 2026 Jan 29;13(2):ofag043. doi: 10.1093/ofid/ofag043 (PMC12923327; doi:10.1093/ofid/ofag043)
Supplement: ofag043_Supplementary_Data [file ofag043_supplementary_data.zip › Supplementary figure 1.docx]

**Supplementary figure 1**


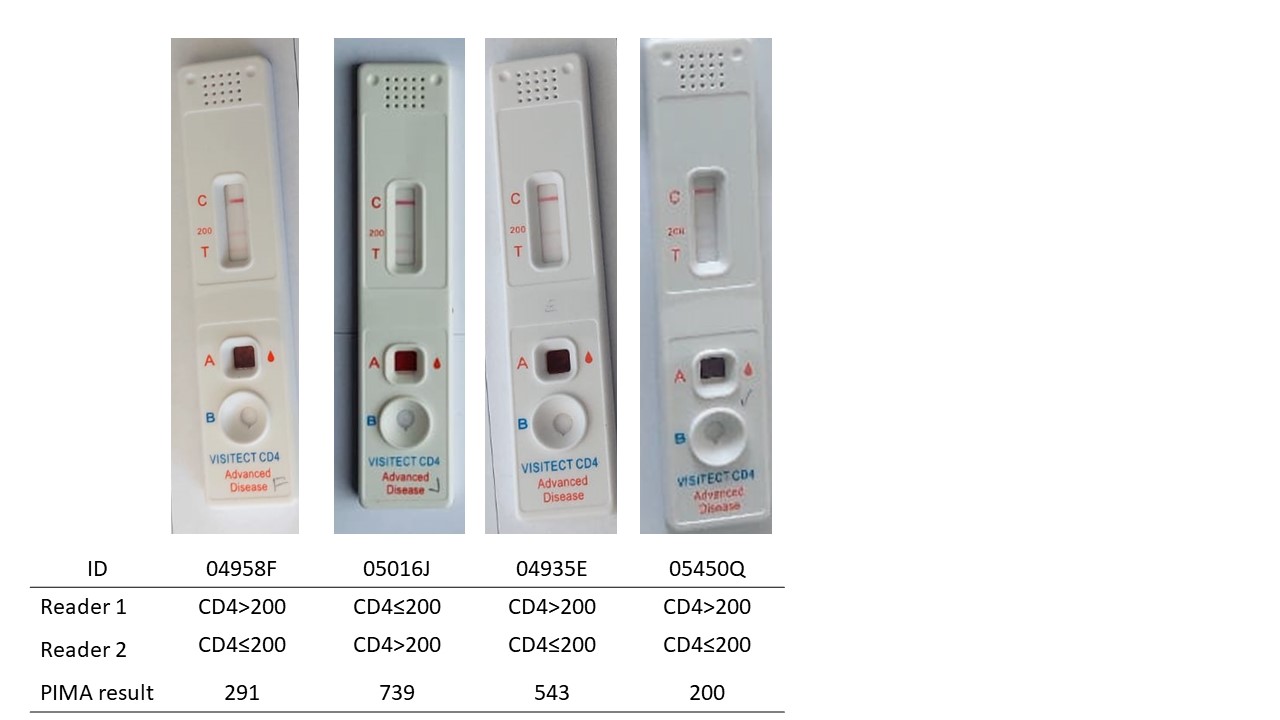


**Examples of VISITECT reading with interrater disagreement**

CD4 is expressed in cells/µl

PIMA; Abbott PIMA CD4 analyser, VISITECT; Accubio VISITECT CD4 Advanced Disease
