# Supplementary material for: Accuracy and Acceptability of the VISITECT CD4 Advanced Disease Test Compared With the PIMA CD4 Test at the Point of Care as Part of the Advanced HIV Disease Care Package: A Mixed-Methods Study
Source: Open Forum Infect Dis. 2026 Jan 29;13(2):ofag043. doi: 10.1093/ofid/ofag043 (PMC12923327; doi:10.1093/ofid/ofag043)
Supplement: ofag043_Supplementary_Data [file ofag043_supplementary_data.zip › Supplementary table 1.docx]

| **Author, year** | **Journal** | **Setting of use** | **Population** | **Reference** | **Sample** | **Operator** | **N** | **Sensitivity [95%CI]** | **Specificity [95%CI]** | **Positive predictive value [95%CI]** | **Negative predictive value [95%CI]** |  |  |  |  |  |  |
| --- | --- | --- | --- | --- | --- | --- | --- | --- | --- | --- | --- | --- | --- | --- | --- | --- | --- |
| Ndlovu et al., 2020 | *PlosONE* | DRC, Malawi, Zimbabwe, laboratory | PWH eligible for CD4 testing | Flow cytometry | Venous blood | Laboratory technician | 708 | 95.0% [91.3 - 97.5] | 81.9% [78.2 - 85.2] | 70.5% [64.9 - 75.6] | 97.3% [95.3 - 98.7] |  |  |  |  |  |  |
|  |  |  | PWH | Flow cytometry | Finger prick | Clinicians | 433 | 98.3% [95.0 - 99.6] | 77.2% [71.6 - 82.2] | NR | NR |  |  |  |  |  |  |
| Lechiile et al., 2022 | *JAIDS* | Botswana, laboratory | PWH >5yrs, baseline CD4 | Flow cytometry | Venous blood | Laboratory technician | 1053 | 94.1% [88.3 - 97.6] | 85.9% [83.5 - 88.0] | 45.9% [39.5 - 52.4) | 99.1% [98.2 - 99.7] |  |  |  |  |  |  |
| Haraka et al., 2024 | *Clin Epidemiol Glob Health* | Tanzania | PWH eligible for CD4 testing | Flow cytometry or PIMA | Finger prick | Laboratory technician, other | 449 | 95.9% [91.8 - 98.3] | 78.3% [73.0 - 83.0] | 73.3% [67 - 79] | 96.9% [93.7 - 98.7] |  |  |  |  |  |  |
|  |  |  |  |  |  | Laboratory technician | NR | 100% [94 - 100] | 84.9% [75.5 - 91.7] | 82.2% [71.5 - 90.2] | 100% [95.1 - 100 ] |  |  |  |  |  |  |
|  |  |  |  |  |  | Other healthcare worker | NR | 93.8% [87.5 - 97.5] | 75.4% [68.7 - 81.3] | 69.1% [61.1 - 76.3] | 95.4% [90.7 - 98.1] |  |  |  |  |  |  |
| Gils et al., 2024 | *JID* | Malawi, South Africa, Tanzania, Thailand, Uganda, Vietnam, Zambia, at POC | PWH | Flow cytometry | Finger prick | Variety of cadres | 1604 | 92.7% [90.1 - 94.7] | 61.4% [58.4 - 64.3] | 53.6% [50.3 - 56.9] | 94.6% [92.7 - 96.2] |  |  |  |  |  |  |
| Nalintya et al., 2024 | *JAIDS* | Uganda, laboratory | PWH entering care | Flow cytometry (if CD4<200)  PIMA or BD Presto (CD4>200) | Venous blood | Laboratory staff | 1495 | 100% [99.2 - 100] | 81.2% [78.7 - 83.5] | 69.1% [65.3 - 72.6] | NR |  |  |  |  |  |  |
| Andreani et al. 2024 | *IJID Regions* | Argentina, laboratory | PWH | FACS calibur | Venous blood | Laboratory staff | 106 | 100% [94.3 - 100] | 18.9% [8.0 - 35.2] | 40% [36 - 44] | 100% [59 - 100] |  |  |  |  |  |  |
| de Haas et al. 2025 | *Trop Med Int Health* | Ethiopia,  Indonesia | PWH eligible for CD4 testing | FACSPresto,  FACSCalibur  PIMA, FACSVia | Venous blood | Laboratory Technicians | 546 | 97.8% [94.4 - 99.4] | 32.7% [27.9 - 37.8] | 41.4% [36.7 - 46.3] | 96.8% [91.9 - 99.1] |  |  |  |  |  |  |
| CI; confidence interval; DRC; Democratic Republic of Congo, PWH; people with HIV, PIMA; Alere PIMA CD4 analyser, POC; point-of-care | | | | | | | | | | | | | |  |  |  |  |
