# Supplementary material for: Accuracy and Acceptability of the VISITECT CD4 Advanced Disease Test Compared With the PIMA CD4 Test at the Point of Care as Part of the Advanced HIV Disease Care Package: A Mixed-Methods Study
Source: Open Forum Infect Dis. 2026 Jan 29;13(2):ofag043. doi: 10.1093/ofid/ofag043 (PMC12923327; doi:10.1093/ofid/ofag043)
Supplement: ofag043_Supplementary_Data [file ofag043_supplementary_data.zip › Supplementary table 2.docx]

**Supplementary table 2: Logistic regression of VISITECT positivity**

| **Variable** | **Categories** | **aOR** |  | **95% CI** | | **p** | |  |
| --- | --- | --- | --- | --- | --- | --- | --- | --- |
| CD4 on PIMA (cells/µl) |  | 1.00* |  | 0.99-1.00* | | 0.000 | |  |
| Age |  | 1.00 |  | 0.97-1.02 | | 0.781 | |  |
| Sex | Female | 1 |  |  | |  | |  |
|  | Male/Ambiguous/Intersex | 1.87 |  | 1.04-3.35 | | 0.036 | |  |
| VISITECT lot number** | X | 1 |  |  | |  | |  |
|  | Y | 6.38 |  | 1.89-21.6 | | 0.003 | |  |
|  | Missing | 1.61 |  | 0.79-3.27 | | 0.188 | |  |
| Days since study start |  | 0.99 |  | 0.99-1.00* | | 0.044 | |  |
| ART status | Known HIV on ART | 1 |  |  | |  | |  |
|  | Known HIV not on ART | 3.07 |  | 0.25-37.5 | | 0.381 | |  |
|  | Newly diagnosed HIV | 1.67 |  | 0.28-10.0 | | 0.577 | |  |
| *rounded up from number < 1.00 | |  |  |  |  | | |  |
| **VISITECT lot number X= 295 (48.4%), Y=95 (15.6%), missing= 219 (36.0%)  ART; antiretroviral treatment, PIMA; Alere PIMA CD4 analyser, VISITECT; Accubio VISITECT CD4 Advanced Disease | | | | | | |  |  |
